# Quantification of within‐ and between‐farm dispersal of *Culicoides* biting midges using an immunomarking technique

**DOI:** 10.1111/1365-2664.12875

**Published:** 2017-02-28

**Authors:** Christopher J. Sanders, Lara E. Harrup, Laura A. Tugwell, Victor A. Brugman, Marion England, Simon Carpenter

**Affiliations:** ^1^ The Pirbright Institute Ash Road Pirbright Surrey GU24 0NF UK

**Keywords:** African horse sickness virus, arbovirus, bluetongue virus, capture–mark–recapture, Ceratopogonidae, epizootic‐haemorrhagic disease virus, ovalbumin, protein marking, Schmallenberg virus, vector‐borne disease

## Abstract

1. *Culicoides* biting midges (Diptera, Ceratopogonidae) are vectors of arboviruses that cause significant economic and welfare impact. Local‐scale spread of *Culicoides*‐borne arboviruses is largely determined by the between‐farm movement of infected *Culicoides*.
2. Study of the dispersal behaviour of *Culicoides* by capture–mark–recapture (CMR) is problematic due to the likelihood of mortality and changes in behaviour upon capture caused by the small size and fragility of these insects, evidenced by low recapture rates. To counter the problem of using CMR with *Culicoides*, this study utilised an ovalbumin immunomarking technique to quantify the within‐ and between‐farm dispersal of *Culicoides* in southern England.
3. Both within‐ and between‐farm dispersal of *Culicoides* was observed. Of the 9058 *Culicoides* collected over 22 nights of trapping, 600 ovalbumin‐positive *Culicoides*, of 12 species including those implicated as arbovirus vectors, were collected with a maximum dispersal distance of 3125 m.
4. This study provides the first species‐level data on the between‐farm dispersal of potential bluetongue, Schmallenberg and African horse sickness virus vectors in northern Europe. High‐resolution meteorological data determined upwind and downwind flight by *Culicoides* had occurred. Cumulative collection and meteorological data suggest 15·6% of flights over 1 km were upwind of the treatment area and 84·4% downwind.
5. *Synthesis and applications*. The use of immunomarking eliminates the potential adverse effects on survival and behaviour of insect collection prior to marking, substantially improving the resolution and accuracy of estimates of the dispersal potential of small and delicate vector species such as *Culicoides*. Using this technique, quantification of the range of *Culicoides* dispersal with regard to meteorological conditions including wind direction will enable improved, data‐driven modelling of the spread of *Culicoides‐*borne arboviruses and will inform policy response to incursions and outbreaks.

## Abstract 2

The use of immunomarking eliminates the potential adverse effects on survival and behaviour of insect collection prior to marking, substantially improving the resolution and accuracy of estimates of the dispersal potential of small and delicate vector species such as *Culicoides*. Using this technique, quantification of the range of *Culicoides* dispersal with regard to meteorological conditions including wind direction will enable improved, data‐driven modelling of the spread of *Culicoides‐*borne arboviruses and will inform policy response to incursions and outbreaks.

## Introduction

*Culicoides* biting midges (Diptera, Ceratopogonidae) are vectors of arboviruses that cause significant economic and welfare impact to the livestock industry, most notably the incursion of multiple serotypes and strains of bluetongue virus (BTV) into Europe (Purse *et al*. 2015). The dispersal of haematophagous arthropod vectors is a key factor in determining the spread of arboviruses (Sellers 1980). Long‐distance dispersal of *Culicoides* overwater has been linked to incursions of *Culicoides*‐borne arboviruses hundreds of kilometres from the nearest virus source and where introduction by movement of viraemic mammalian hosts was discounted (Sellers, Pedgley & Tucker 1978; Alba, Casal & Domingo 2004; Burgin *et al*. 2012). In contrast, studies examining overland spread of BTV by correlating wind‐density maps and outbreak data (Hendrickx *et al*. 2008) and stochastic simulation of likely time of infection and wind speeds and direction (Sedda *et al*. 2012) have found limited evidence for long‐distance dispersal of *Culicoides*. In both studies, the model prediction of distance for arbovirus spread was less than 5 km day^−1^ and was largely determined by the between‐farm movement of infected *Culicoides* (Hendrickx *et al*. 2008; Sedda *et al*. 2012). Quantification of the range and probability of *Culicoides* dispersal within‐ and between‐farms is essential to allow accurate estimation of the speed and extent of spread of *Culicoides‐*borne arboviruses and to inform policy response during incursions and outbreaks (Defra 2014).

A variety of methods have been used to quantify *Culicoides* dispersal at a local scale. Movement from isolated areas of larval habitat has been used where these can be readily identified and demarcated (Kettle 1960; Williams 1962; Zimmerman & Turner 1984). *Culicoides* have also been marked using of radioactive isotopes (Davies 1965; Holbrook, Belden & Bobian 1991) and ingestion of food dye (Campbell & Kettle 1976), however these techniques utilised emerging adults and are thus reliant on the availability of larval habitat. The putative vectors of BTV in the northern Palaearctic; *Culicoides obsoletus* Meigen, *Culicoides scoticus* Downes and Kettle, *Culicoides dewulfi* Goetghebeur, *Culicoides chiopterus* (Meigen), *Culicoides pulicaris* (L.) and *Culicoides punctatus* (Meigen) (Hoffmann *et al*. 2009; Elbers *et al*. 2013) utilise almost ubiquitous farm‐associated larval habitats unsuitable for these techniques (Harrup *et al*. 2013). Capture–Mark–Recapture (CMR) of adult *Culicoides* has also been carried out using fluorescent dusts (Lillie, Jones & Marquardt 1981; Lillie, Kline & Hall 1985; Linhares & Anderson 1989; Carpenter 2001). The mean dispersal distance of *Culicoides* in these studies was found to be limited to a few hundred meters in most cases, with the exception of a few individuals making flights of 2–3 km, and up to 6 km in the dessert‐adapted *Culicoides mohave* Wirth (Brenner *et al*. 1984).

Two recent studies have examined the dispersal of *Culicoides* in northern Europe. An estimated 61 000 *Culicoides* marked with fluorescent dust were released by Kluiters, Swales & Baylis (2015), of which 0·02% (12) were recaptured at distances between 1 and 2·5 km from the release site, suggesting limited between‐farm movement. Kirkeby *et al*. (2013) utilised a fluorecin isothiocyanate dye to mark an estimated 1460 *Culicoides*, of which 3·28% (48) were recaptured. Of these, two *Culicoides* were recaptured at the point‐of‐release trap site, while 28 specimens were recaptured at a distance of up to 1750 m from the release site, supporting between‐farm movement. However, the low number of individuals recaptured in these studies, the lack of detailed meteorological recording and identification to group rather than species level compromises attempts to accurately quantify the dispersal of Palaearctic *Culicoides* within a species group already known to differ in seasonality, host and habitat use at the species level (Searle *et al*. 2013).

A key assumption of CMR studies is that the marking process has no effect on the survival and behaviour of the individual (Hagler & Jackson 2001). The small size (<3 mm wingspan) and fragility of smaller Diptera of medical and veterinary importance, including the Ceratopogonidae (biting midges) and Phlebotominae (sand flies) present a significant challenge. *Culicoides* collected overnight in light‐suction traps prior to marking (Kirkeby *et al*. 2013; Kluiters, Swales & Baylis 2015), are visibly dehydrated, experience significant mortality and often exhibit inhibited host‐feeding behaviour (Venter *et al*. 2005).

An alternative technique that addresses some of the limitations of CMR is the use of protein‐based immunomarking. Insects are exposed to a protein marker, either by direct application or indirectly via a discrete marked area of habitat through which they pass (Hagler *et al*. 1992). Insects are then collected and an enzyme‐linked immunosorbent assay (ELISA) used to detect the marker, allowing cost‐effective and rapid screening of thousands of individuals (Jones *et al*. 2006). The technique has been successfully used to mark pollinator, phytophagous and predaceous insects (Hagler 2011; Biddinger *et al*. 2013; Swezey *et al*. 2013). Immunomarking has also been used on *Anaphes iole* Girault (Hymenoptera: Mymaridae), minute parasitoid wasps that at 1 mm in length are smaller even than *Culicoides* (Hagler & Jackson 1998). Sanders & Carpenter (2014) have previously demonstrated that an ovalbumin marker persisted on *Culicoides nubeculosus* (Meigen) for 72 h post‐exposure with no detrimental effects on survival. A preliminary field trial also demonstrated that ovalbumin‐marked subgenus *Avaritia Culicoides* could be recovered from areas close (≤20 m) to marked larval development habitat (Sanders & Carpenter 2014). In this study, we utilise the ovalbumin immunomarking technique for the first time at a broader‐scale to quantify the within‐ and between‐farm dispersal of *Culicoides* in southern England.

## Materials and methods

### Study site and habitat immunomarking

**Figure 1:**
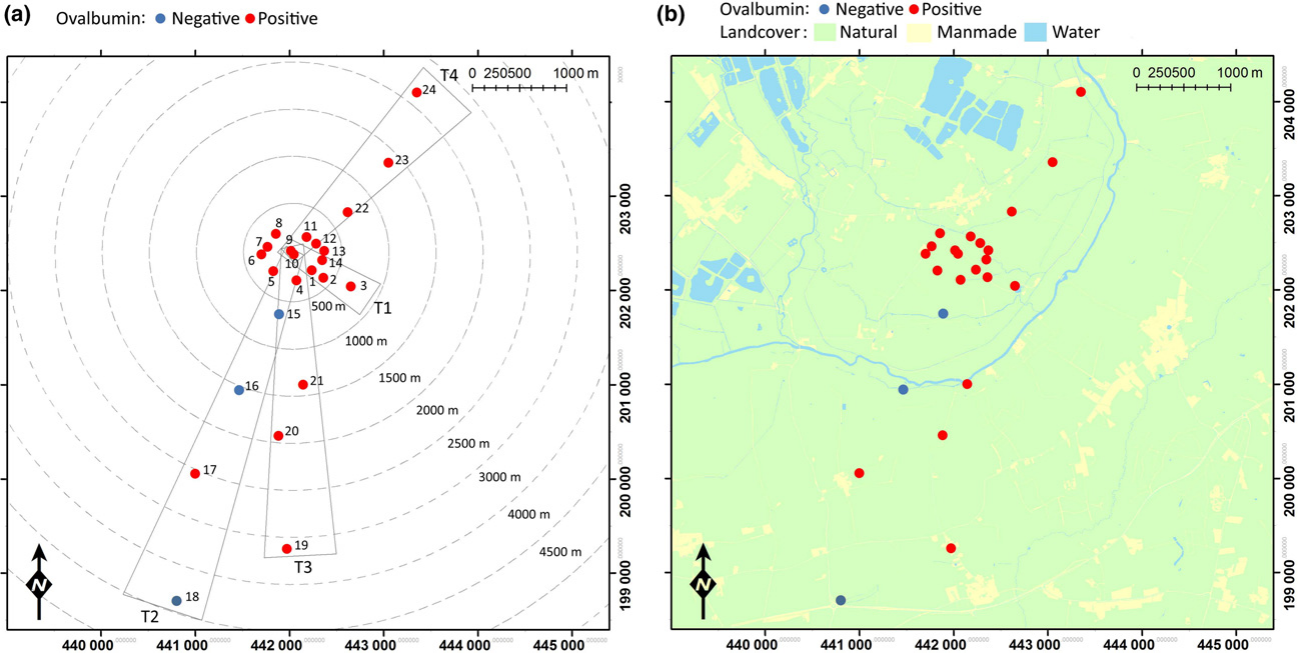
Trap locations, transect areas (a) and geographical location of study site (b). Colour of trap location indicates whether ovalbumin‐positive *Culicoides* were collected at that location at any point during the study. Traps 1–14 were used in 2013, traps 1–24 were used in 2015. [Colour figure can be viewed at wileyonlinelibrary.com]

**Figure 2:**
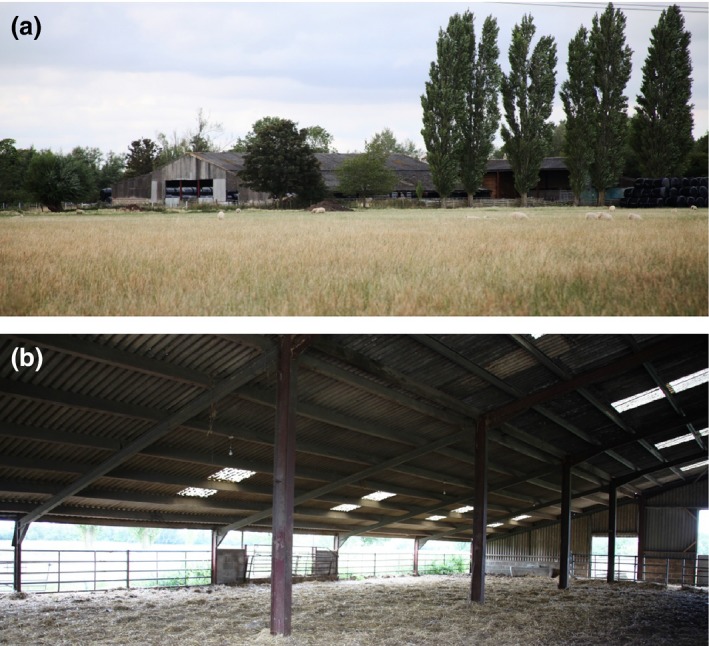
External (a) and internal (b) view of cattle barn. Cattle barn utilised for habitat immunomarking. [Colour figure can be viewed at wileyonlinelibrary.com]

Habitat immunomarking was conducted at a mixed sheep and beef cattle farm in southern England (51°43′39N; 1°23′16W) (Fig. 1a). Surrounding the farm were additional livestock holdings to the south and east, with arable crops to the west and a residential area and water reservoirs to the north (Fig. 1b). Two areas (total of ~60 m^2^) of mixed cattle dung and straw bedding along two sides of an open‐sided shed (Fig. 2) were sprayed 2–3 h prior to sunset on day 0 using a hand‐operated knapsack sprayer (Silverline, Yeovil, UK) with 40 L of 20% (w/v) liquid egg white solution (Sports Supplements Ltd, Colchester, UK), resulting in an approximate application rate of 0·33 Lm^−2^. Insects were then collected for 3 or 4 days post‐treatment. To prevent cross‐contamination, different personnel and equipment were used to mark habitat to those responsible for insect collection. Five egg solution treatments were conducted; two during 2013 and three during 2015, with a minimum period of 2 weeks between the last day of insect collection and the next treatment.

### Meteorological conditions

Meteorological conditions [temperature (°C), wind speed (ms^−1^), wind direction (°), relative humidity (%), solar radiation (Wm^−2^) and precipitation (mm)] were monitored at 1–15 min resolution using an automatic weather station located 50 m from the marked area and analysed using the openAir (Carslaw & Ropkins 2012) package in R v. 3.2.2 (R Development Core Team 2015) with RStudio v. 0.99.467 (RStudio Team 2015).

### *Culicoides* collection

Insect collections were completed overnight using ultraviolet miniature Center for Disease Control traps (model: 912; John W Hock, Gainsville, FL, USA) suspended at approximately 1·2 m from metal hooks (Gardman Ltd, Peterborough, UK). All traps were located at field margins and hedgerows beyond the reach of livestock. Insects were collected dry into disposable, single use 560 mL polypropylene cups that contained a small quantity of shredded paper towel based on a preliminary study to minimise reduce contact and therefore potential marker transfer between collected specimens (Fig. S1, Supporting Information). Collected insects were immobilised by exposure to cold (−20 °C) and subsequently stored at −20 °C prior to further analysis.

Fourteen trap locations were used in two trial replicates completed in 2013 (locations 1–14, Fig. 1). Trap locations two and three formed a short linear transect (T1) extending southeast from the marked area (Fig. 1). In three replicates in 2015, the 14 trap locations utilised in 2013 were supplemented with an additional 10 trap locations which formed three linear transects extending out from the egg‐marked area to a maximum distance of 3876 m (Fig. 1). These three transects extended through the livestock pastures of adjacent farms and represent potential dispersal routes to the available livestock hosts of dairy cattle (T2), beef cattle (T3) and sheep (T4) respectively.

### Ovalbumin‐specific ELISA

An ovalbumin‐specific ELISA modified from Hagler & Jones (2010) was utilised to detect ovalbumin‐positive *Culicoides*. Ovalbumin‐positive controls were created by either soaking a 225 mm^2^ piece of paper, which had been dipped in egg white solution, in 300 μL of tris buffered saline (TBS) (pH 7·4) (Alfa Aesar, Heysham, UK) with 0·001% Silwet L‐77 (De Sangosse Ltd, Cambridge, UK) (TBS‐SILWET) (Sanders & Carpenter 2014) or via the addition of 3 μL egg white solution per 300 μL TBS‐SILWET. From this, a 10‐fold serial dilution was created in order to monitor relative detection sensitivity between assay runs. *Culicoides* were identified morphologically to species or subgenus/group level using the keys of Campbell & Pelham‐Clinton (1960) and reference wing images (The Pirbright Institute 2007). *Culicoides* identified from their morphology were then transferred individually using disposable cocktail sticks to tubes containing 300 μL TBS‐SILWET and incubated at 4 °C overnight.

Subsequently, 80 μL of the sample solutions were transferred to the corresponding wells of an ELISA plate. Negative controls (80 μL TBS‐SILWET) and positive controls (10^0^, 10^−1^, 10^−2^, 10^−3^ and 10^−4^ dilutions) were then added to the remaining wells of the ELISA plate. The ELISA plate was then incubated at 37 °C for 1 h; washed five times with phosphate‐buffered saline with tween‐20 (PBST) (Sigma‐Aldrich, Gillingham, UK); 360 μL of phosphate‐buffered saline with bovine serum albumin (PBS‐BSA) (Sigma‐Aldrich) added per well; incubated at room temperature for 1 h; washed twice with PBST; 80 μL 0·0125% rabbit anti‐chicken egg albumin (Sigma‐Aldrich) in PBS‐BSA with 0·13% (w/v) Silwet L‐77 (PBS‐BSA‐SILWET) added per well; incubated for 1 h at 37 °C; washed five times using PBST; 80 μL of 0·05% goat anti‐rabbit IgG conjugated to horseradish peroxidase (Sigma‐Aldrich) in PBS‐BSA‐SILWET added per well; incubated for 2 h at 37 °C; washed five times using PBST; 80 μL of 1‐Step Ultra TMB‐ELISA Substrate solution (ThermoFisher Scientific, Warrington, UK) added per well; incubated at room temperature for 10 min followed by the addition of 80 μL 2N sulphuric acid (Sigma‐Aldrich) to stop the reaction. The ELISA optical density (OD) of each well was measured at 450 nm with a microplate reader.

All ODs were transformed using the standard normal variate (SNV) transformation (Sivakoff, Rosenheim & Hagler 2011). The threshold score was set using the ‘maximum negative control’ algorithm i.e. the highest negative control score observed across all ELISA plates in the study (Hooper & Woolson 1991; Sivakoff, Rosenheim & Hagler 2011). Specimens were classified as ovalbumin‐positive if their score was greater than the selected threshold.

### *Avaritia* subgenus species identification

Following the ovalbumin‐specific ELISA, subgenus *Avaritia* specimens collected in replicates 3–5 were transferred individually to 200 μL of 5·0% Chelex^®^ 100 resin (Bio‐Rad, Hemel Hempstead, UK) solution with 5·0% proteinase K (Qiagen Ltd, Manchester, UK); homogenised at 25 Hz for 4 min using a tissue‐lyser (Qiagen Ltd) and the homogenate incubated overnight at 37 °C to extract total DNA. A multiplex polymerase chain reaction (PCR) assay was used then to identify samples to species level (Nolan *et al*. 2007). Amplification reactions were performed in a total of 20 μL consisting of 0·6 μL nuclease free water, 10·0 μL Qiagen^®^ Fast Cycing PCR Mastermix (Qiagen Ltd), 2·0 μL CoralLoad concentrate (Qiagen Ltd), 2·0 μL 50% D‐(+)‐Trehalose (Sigma‐Aldrich), 0·4 μL 50 mM magnesium chloride (Qiagen Ltd), 40 mM forward primers: 0·1 μL UOAchiF, 0·1 μL UOAdewF, 0·1 μL UOAobsF and 0·2 μL UOAscoF (Nolan *et al*. 2007) and 0·5 μL 40 mM reverse primer C1‐N‐2191 (Dallas *et al*. 2003) and 4 μL Chelex supernatant (DNA template). Positive and negative controls for the amplification reactions were carried out at every PCR round. The PCR thermal cycle included: an initial denaturation step at 95 °C for 5 min followed by 35 cycles of 96 °C for 5 s, 48 °C for 5 s, 72 °C for 15 s, followed by a final extension step at 72 °C for 1 min. Amplification success and banding patterns were assessed by electrophoresis of PCR products on 2% (w/v) pre‐cast agrose gels containing SYBR^®^ Safe (E‐Gel^®^ 96; ThermoFisher Scientific).

### Statistical analysis

Generalised linear mixed models (GLMM) with a Binomial error distribution and a logit link function were used to investigate the influence of meteorological conditions and the spatial relationship between trap location and the egg‐marked area in determining the presence of ovalbumin‐positive *Culicoides* within‐trap catches. Models were implemented in a Bayesian setting using the bglmer function in package ‘blme’ v. 1.0‐2 (Dorie 2014) in R v. 3.1.2 (R Development Core Team 2015) with RStudio v. 0.99.467 (RStudio Team 2015).

The GLMMs were fitted by maximum likelihood with the Laplace approximation with flat covariance priors and normal fixed priors, with *days since egg marking* included as a random effect, and a total of five additional fixed predicators including: *distance from egg‐marked area*,* difference between trap bearing and mean wind direction*,* mean air temperature*,* mean relative humidity*,* mean precipitation*,* mean solar radiation*,* mean wind speed*,* maximum gust speed* as linear functions were considered. The fixed predictors were zero‐centred and scaled to yield a standard deviation of one. Final models were obtained using a backwards‐stepwise‐selection‐based procedure (Zeileis, Kleiber & Jackman 2008), such that variables that did not contribute significantly to explaining variation in trap catch were successively eliminated on the basis of Akaike information criterion (AIC) (Akaike 1973). This continued until the removal of a variable caused an increase in AIC of two or more.

## Results

**Figure 3:**
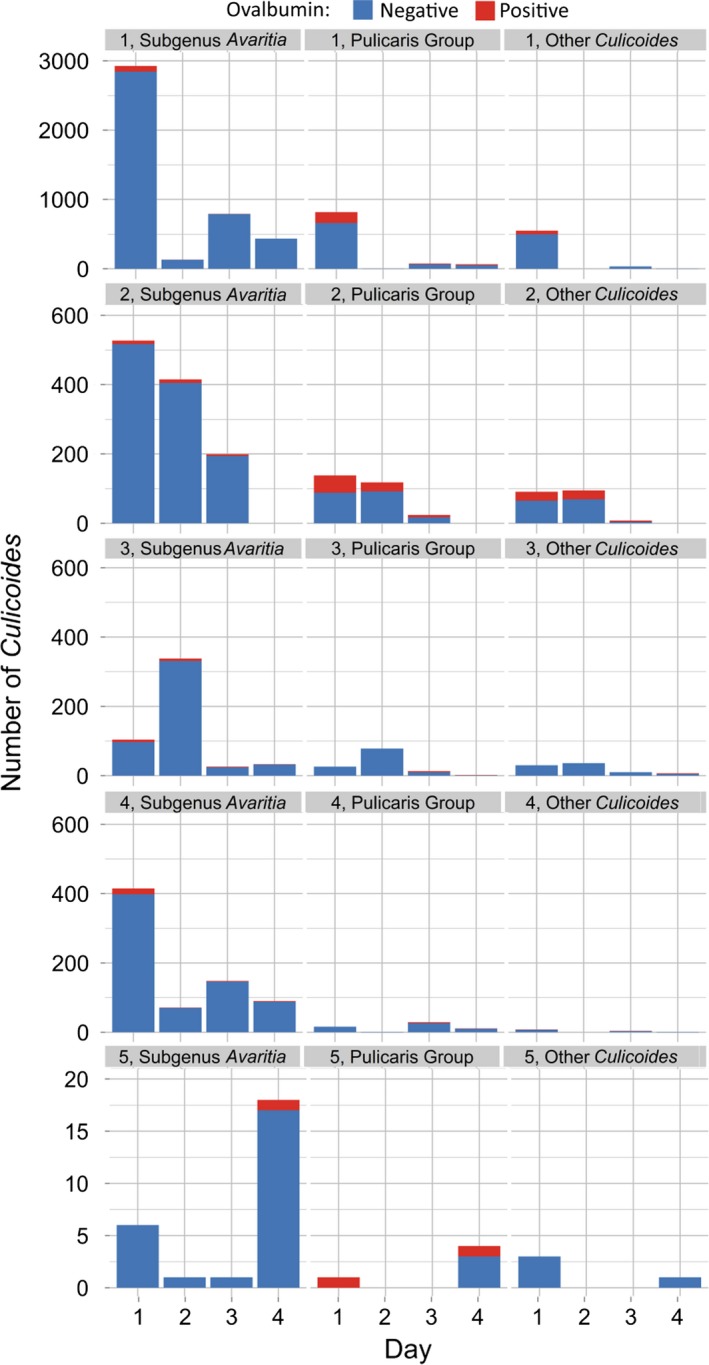
Number of *Culicoides* collected per day post‐treatment for each of five replicates of the immunomarking trial. Data are split by species group and the number of ovalbumin‐positive individuals recovered. [Colour figure can be viewed at wileyonlinelibrary.com]

**Figure 4:**
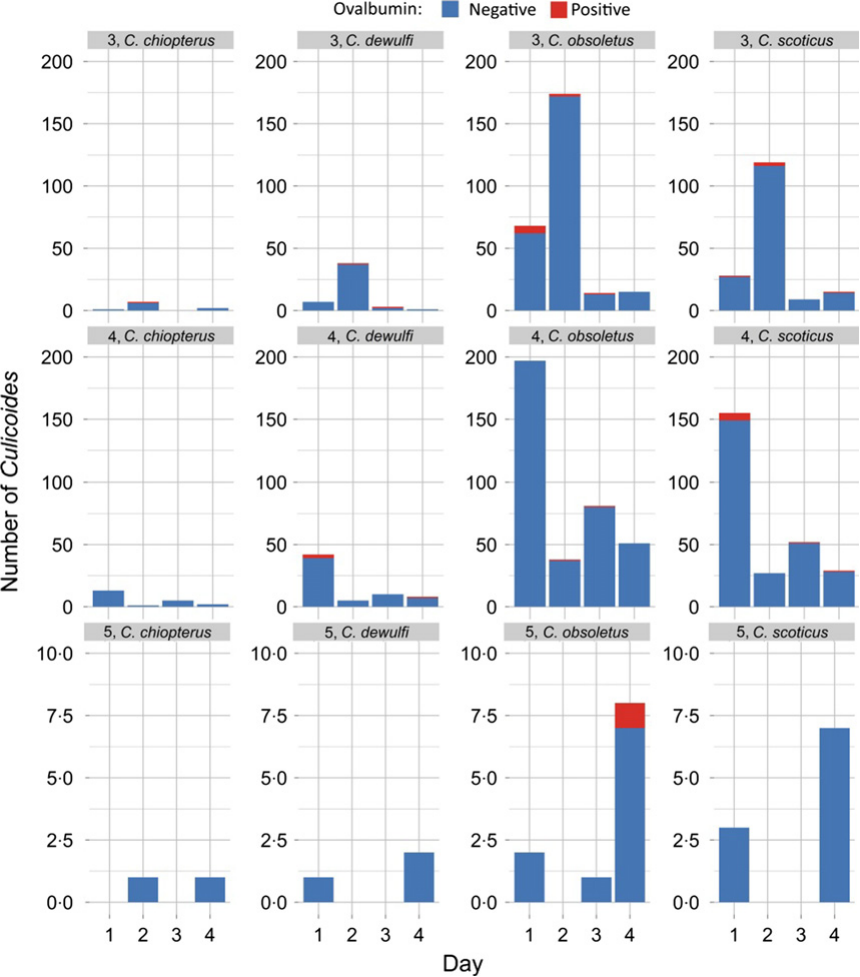
Number of subgenus *Avaritia Culicoides* collected per day post‐treatment for replicates three, four and five of the immunomarking trial. Data are split by species and the number of ovalbumin‐positive individuals recovered. [Colour figure can be viewed at wileyonlinelibrary.com]

A total of 9058 *Culicoides* were collected over 22 nights of trapping (Fig. 3, Table S1). In 2013, 140 trap collections were completed with 7526 *Culicoides* collected from 14 trap locations over 10 nights of trapping. In 2015, 288 trap collections were completed with 1532 *Culicoides* collected from 24 trap locations over 12 nights of trapping (Table S1). *Culicoides* collections were dominated by female *Culicoides* with only 1% of *Culicoides* collected being male (Table S1). In total, 14 *Culicoides* species were identified: *Culicoides achrayi* Kettle and Lawson, *Culicoides albicans* (Winnertz), *C. chiopterus*,* Culicoides circumscriptus* Kieffer, *Culicoides clastrieri* Callot, Kremer and Deduit, *C. dewulfi*,* Culicoides festivipennis* Kieffer, *C. nubeculosus*,* C. obsoletus*,* Culicoides pictipennis* (Staeger), *C. pulicaris*,* C. punctatus*,* Culicoides riethi* Kieffer and *C. scoticus* (Figs 3 and 4, Table S1). Trap catches were dominated by subgenus *Avaritia* species, which accounted for 74·0% of the total number of *Culicoides* collected. Of the other *Culicoides* species collected, *C. punctatus*,* C. pulicaris*,* C. achrayi* and *C. festivipennis* were the most abundant in trap catches accounting for 8·6%, 7·1%, 6·9% and 2·9% of the total number of *Culicoides* collected, respectively (Table S1). The remaining six species accounted for only 0·6% of the total number of *Culicoides* collected (Table S1). Subgenus *Avaritia* specimens collected in replicates three, four and five, which were identified to species level were dominated by *C. obsoletus* (52·5%), with 35·5%, 9·4% and 2·6% *C. scoticus, C. dewulfi* and *C. chiopterus* collected respectively (Fig. 4, Table S1).

**Figure 5:**
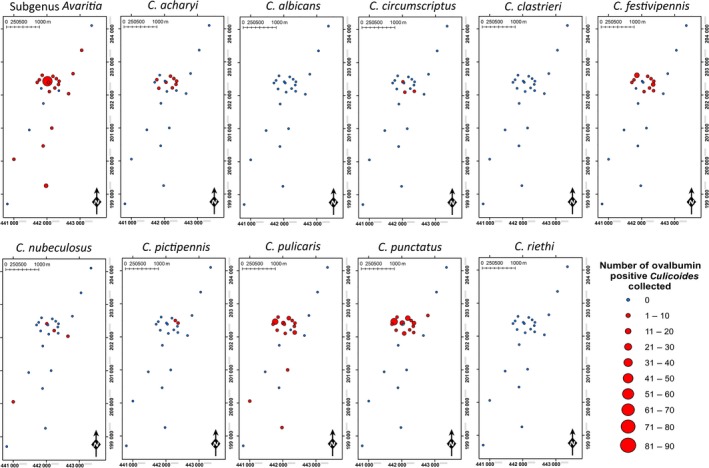
Spatial distribution of ovalbumin‐positive *Culicoides* collected (replicate 1–5). [Colour figure can be viewed at wileyonlinelibrary.com]

**Figure 6:**
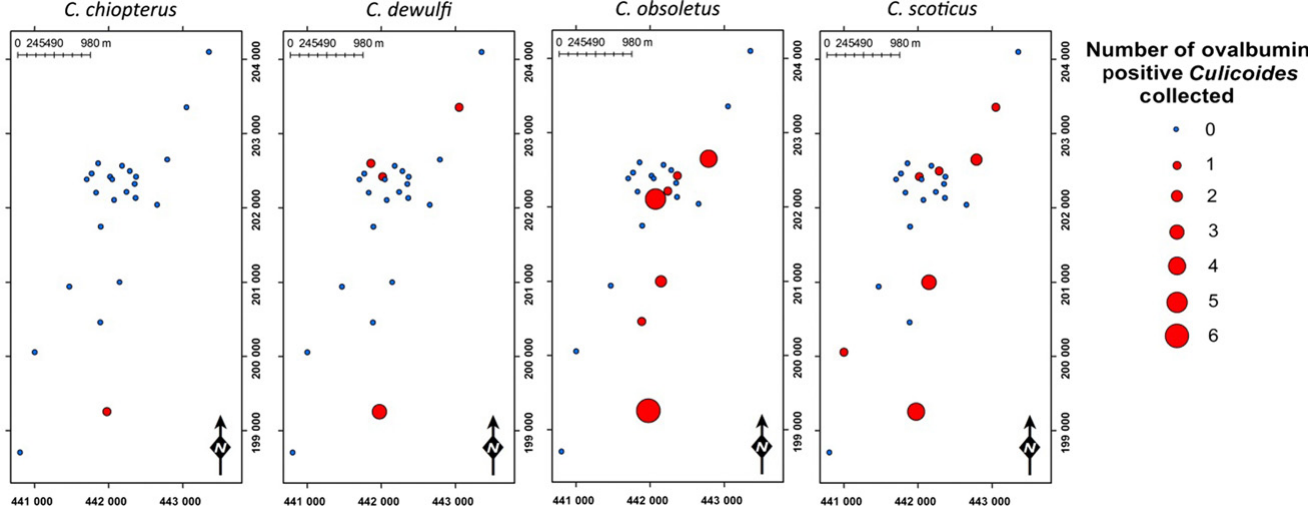
Spatial distribution of ovalbumin‐positive subgenus *Avaritia Culicoides* collected during replicates 3–5. [Colour figure can be viewed at wileyonlinelibrary.com]

A total of 600 *Culicoides* from ten species were observed to be positive for ovalbumin; *C. achrayi*,* C. chiopterus*,* C. dewulfi*,* C. circumscriptus, C. festivipennis*,* C. nubeculosus*,* C. obsoletus*,* C. pulicaris, C. punctatus* and *C. scoticus* (Figs 3 and 4, Table S2). The maximum distance from the egg‐marked area that *Culicoides* were collected and observed to be ovalbumin‐positive was 3125 m (Table 1, Figs 5 and 6). Ovalbumin‐positive Subgenus *Avaritia Culicoides* were observed at the maximum distance of 3125 m from the egg‐marked area as early as day 1, however positive *C. pulicaris* individuals were not collected at this distance until day 3 (Table 1). In replicates three, four and five of the trial where subgenus *Avaritia* individuals were identified to species level, *C. dewulfi*,* C. obsoletus* and *C. scoticus* were recorded at 3125 m at day 1; however, *C. chiopterus* was not recorded at this distance until day 2 (Table 1). Ovalbumin‐positive *C. nubeculosus* individuals were collected at a maximum distance of 2542 m by day 4, and *C. punctatus* individuals at a maximum of 710 m by day 3. Ovalbumin‐positive specimens from all other species were collected ≤406 m from the egg‐marked area (Table 1). All traps at a distance of <500 m collected ovalbumin‐marked individuals. In 2015, all transects extending beyond 1 km had traps that collected positive individuals, with the greatest number collected along transect three (Figs 5 and 6, Table S2).

**Table 1:** Maximum dispersal distance of collected *Culicoides* by day and species/species group (– indicates specimens positive for ovalbumin not collected on this day in any replicate or trap catch)

Species	Maximum observed dispersal distance (m)			
	Day 1	Day 2	Day 3	Day 4
Subgenus *Avaritia Culicoides* species^a^	3125	3125	3125	3125
* Culicoides chiopterus* b	–	3125	–	–
* Culicoides dewulfi* b	3125	247	1377	6
* Culicoides obsoletus* b	3125	1385	3125	3125
* Culicoides scoticus* b	3125	1385	3125	1377
*Culicoides acrayi*	314	–	314	–
*Culicoides circumscriptus*	406	–	–	–
*Culicoides festivipennis*	406	314	314	–
*Culicoides nubeculosus*	698	–	6	2542
*Culicoides pictipennis*	314	–	–	–
*Culicoides pulicaris*	406	406	3125	3125
*Culicoides punctatus*	406	307	710	6

a *Based on data from replicates 1–5. Includes number of *Culicoides* displayed in *C. chiopterus*,* C. dewulfi, C. obsoletus* and *C. scoticus* rows.

b Species identifications based on multiplex PCR assay of specimens collected in replicates three, four and five only.

**Figure 7:**
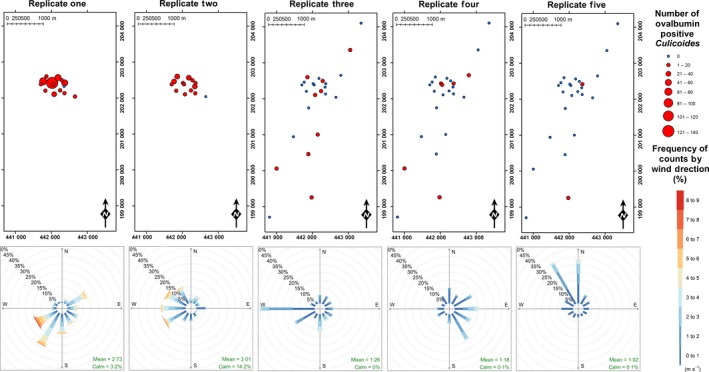
Locations of ovalbumin‐positive *Culicoides* collections and windroses displaying cumulative mean windspeed (ms^−1^) as a function of wind direction (°) summarised by replicate. [Colour figure can be viewed at wileyonlinelibrary.com]

*Mean air temperature, mean relative humidity, mean solar radiation, mean wind speed* and *distance from the egg‐marked area* were all found to be significant determinants of the likelihood of ovalbumin‐positive *Culicoides* being collected (Table 2). Within the range of meteorological conditions experienced during this study (Fig. 7, Table S3), increasing *mean air temperature* (*P* ≤ 0·001), *mean relative humidity* (*P* ≤ 0·01) and *mean solar radiation* (*P* ≤ 0·01) were found to significantly increase the likelihood of ovalbumin‐positive *Culicoides* being collected within a trap catch (Table 2). Increasing *mean wind speed* (*P* ≤ 0·001) and increasing *distance from the egg‐marked area* (*P* ≤ 0·01) were found to significantly decrease the likelihood of ovalbumin‐positive *Culicoides* being collected within a trap catch (Table 2).

**Table 2:** Regression coefficients with 95% Wald confidence intervals and ∆AIC for the fixed effects of the final Bayesian general linear mixed models with a Binomial error distribution used to describe the presence of ovalbumin‐marked *Culicoides* (all species) (random effects: *days since egg treatment*)

Parameters	Estimate (95% CI)	∆AIC
Intercept	−3·48 (−4·39 to −2·57)***	
Mean air temperature	2·83 (1·50–4·17)***	18·34
Mean relative humidity	1·33 (0·43–2·22)**	5·71
Mean solar radiation	2·13 (0·81–3·47)**	9·04
Mean wind speed	−2·30 (−3·63 to −0·97)***	12·22
Distance from egg‐marked area	−1·73 (2·85 to −0·62)**	9·66

**P ≤ 0·01, ***P ≤ 0·001.

Wind direction was not found to be a significant determinant of the probability of collecting ovalbumin‐positive *Culicoides*, demonstrated by the observation of both upwind and downwind flight from the treatment area (Fig. 7). Of the ovalbumin‐positive *Culicoides* collected at more than 1 km from the treatment site on the first trapping session of each replicate, one was observed to have flown upwind and 11 downwind. Subsequent collections were not independent of the wind direction of previous sessions within the replicate, and therefore flight direction cannot be determined but cumulative data for wind direction and collection show that of individuals collected at distance greater than 1 km from the treated area, 15·6% were upwind and 84·4% downwind of the treated area (Fig. 7).

In addition to *Culicoides*, 27 female *Culex* mosquitoes were collected of which 18 were found to be positive for ovalbumin from trap locations two, three, four, five, six, seven, 10 and 13. Ovalbumin‐positive *Culex* were collected up to a maximum of 698 m on day 3 from the egg‐marked area, the maximum distance from the egg‐marked area ovalbumin‐positive *Culex* was recorded on day 1 post‐egg‐spraying was 314 m.

## Discussion

This study uses a habitat marking approach to provide the first, species‐level data on the within‐ and between‐farm dispersal of Palaearctic *Culicoides*. Previous estimates of the dispersal potential of Palaearctic *Culicoides* had been made from CMR studies with very limited data from the recapture of a few marked individuals (Kirkeby *et al*. 2013; Kluiters, Swales & Baylis 2015). In contrast, this study used an immunomarking technique to mark insects in and around a potential larval development site and collected 600 marked *Culicoides*.

The majority (94%) of marked *Culicoides* were collected within 1 km of the treatment area, where trap density was greatest; suggesting most individuals which had emerged from or visited the marked habitat subsequently engaged in short appetitive flights to locate hosts, mates or breeding habitat. The fact that *Culicoides* were intercepted within traps at close range before they were able to disperse may lead to an underestimation of the potential for long‐distance movement. Despite the low density of traps, 6% of the ovalbumin‐positive insects were collected in traps 1·3–3·1 km from the marked area, with *C. dewulfi, C. obsoletus* and *C. scoticus* demonstrating a maximum‐recorded dispersal distance of more than 3 km over just one night. Ovalbumin‐positive *Culicoides* were collected from all three transects, in areas used for grazing both cattle and sheep, with the greatest number collected in the trap near the greatest density of cattle suggesting individuals were engaged in active host‐seeking behaviour. *Culicoides pulicaris* and *C. nubeculosus* individuals were collected at the maximum distance only 3 or 4 days post‐marking, with *C. punctatus* and other species not recorded at distances greater than a kilometre from the marked area. This contrasts with the previous dispersal data of Pulicaris group where individuals were recaptured at 1750 m from the release site two or three nights after release (Kirkeby *et al*. 2013). This may reflect the lower abundance of these species in this study area and therefore the reduced probability of a marked individual being captured. Topographical features of the study area including hedgerows, trees and a river did not appear to influence the direction of *Culicoides* dispersal. The within‐ and between‐year variation in difference in *Culicoides* abundance at the site may be due to increased drying of the larval habitat in the summer of 2015 in comparison with 2013 when the site had been flooded over the winter.

In previous studies, the low number of individuals recaptured (40 in total) at all distances obscures the estimation of local movement of the majority of *Culicoides* individuals (Kirkeby *et al*. 2013; Kluiters, Swales & Baylis 2015). Using the SNV transformation of ELISA data did not affect the maximum dispersal distance of marked *Culicoides*. It did, however, have a significant effect on the proportion of trap catch recorded as positive for ovalbumin marker, with 27·1% and 6·8% positive using the conventional threshold and SNV transformation respectively (Table S4). Whilst this may result in a conservative calculation of the number of marked individuals caught, the data are more robust and can be used with greater confidence.

Whilst the immunomarking technique used within this study offers considerable insights into behaviours that were previously precluded by the small‐size and fragility of *Culicoides*, the technique does have limitations. The potential transfer of the marker protein between marked and unmarked individuals in a trap catch is of concern as a source of false positives (Hagler, Machtley & Blackmer 2015). When tested in the laboratory under optimal conditions, the transfer of ovalbumin between colony *C. nubeculosus* was limited, with evidence of transfer in only one replicate at a high ratio of marked to unmarked individuals (15 marked : 50 unmarked) (Fig. S1). In this study, marker transfer between *Culicoides* would not affect interpretation of the distance travelled by marked insects. However, in studies where the number of marked individuals would have a significant bearing on interpretation of results, the potential for marker transfer should be considered.

As the number of individuals marked is unknown, the proportion of the population that engaged in dispersal and estimates of survivorship and migration (Lysyk & Axtell 1986; Hanski, Alho & Moilanen 2000) cannot be estimated from the data in this study. For arbovirus vectors, even rare dispersal events may have significant impacts on epidemiology. The 6% of the marked individuals collected on adjacent pastures at greater than 1 km imply *Culicoides* on lowland farms represent well‐connected populations with significant mixing between farms. Within replicates three to five, traps at a distance greater than 1 km collected 59% of marked individuals. The rapid spread of BTV and particularly SBV across northern Europe (Carpenter, Wilson & Mellor 2009; Elbers *et al*. 2012; Balenghien *et al*. 2014) has been used to infer a substantial movement of *Culicoides* between farms. The present data support previous estimates of dispersal distance of 3–5 km per day of infected *Culicoides* (Ducheyne *et al*. 2007; Hendrickx *et al*. 2008; Sedda *et al*. 2012; Sedda & Rogers 2013). There is, however, evidence of population‐scale differences in phylogeny in *Culicoides* (Jacquet *et al*. 2016) that suggests the presence of barriers to the dispersal of *Culicoides* at a greater scale than represented in this study.

Collection of ovalbumin‐positive *Culicoides* was not significantly influenced by wind direction, with upwind and downwind flight observed in both cumulative and session‐by‐session analysis of collections and wind direction. Since collection may not occur at the night during which dispersal occurred, only the first trapping session can be used to confirm upwind or downwind flight of individuals, and here we present evidence for upwind flight and downwind flight of individual *Culicoides* to distances greater than 1 km in a 24 h period. Whilst Kluiters, Swales & Baylis (2015) suggest upwind and downwind flight from the release point occurred in their study, the wind speed and direction used data were at too low a resolution (24 h average) to test this hypothesis given the likely influence of the local topographic complexity on wind direction (Whiteman & Doran 1993). Here, all replicates took place under favourable conditions for *Culicoides* flight, with light winds (<3 ms^−1^) (Fig. 7). Increasing wind speed increased the likelihood of capture at distance from the marked area, suggesting downwind transport may also play a role in local dispersal. Increased lunar radiation correlated with increased probability of detecting ovalbumin‐positive *Culicoides* likely due to the increased *Culicoides* activity on moonlit nights (Linhares & Anderson 1990; Bishop *et al*. 2000).

## Conclusion

The ovalbumin‐immunomarking technique has been demonstrated to be a highly effective tool for the study of *Culicoides* dispersal behaviour with evidence it may be suitable for other hematophagous vectors e.g. *Culex* and Phlebotominae. The use of the immunomarking technique in areas of increased topographical complexity, potentially involving multiple marker proteins to label different hosts or habitats within a single location (Hagler & Jones 2010), combined with investigations of gene flow between populations using landscape genetics (Manel & Holderegger 2013) and deep sequencing of viral genomes, could illustrate how *Culicoides* and the arboviruses they transmit move through the environment and quantify the relative impact of barriers to dispersal. Increased confidence in the estimates of within‐ and between‐farm dispersal of *Culicoides* will enable improved data‐driven modelling of the spread of *Culicoides*‐borne arboviruses (Graesboll *et al*. 2016) and will inform policy response to incursions and outbreaks at both the national and transnational level.

## Authors' contributions

C.J.S. and L.E.H. collected the data, S.C., L.T., V.B. and M.E. assisted in fieldwork; L.E.H. analysed the data; C.S. and L.E.H. led the writing of the manuscript. All authors contributed critically to the drafts and gave final approval for publication.

## Data accessibility

Data used in the production of this manuscript are available from the Dryad Digital Repository https://doi.org/10.5061/dryad.s403s (Sanders *et al*. 2017).

## Supporting information


**Table S1.** Distance (m) of trap locations relative to the egg solution‐marked area and number of *Culicoides* collected.

**Table S2.** Number of *Culicoides* collected positive for ovalbumin during immune‐marking studies.

**Table S3.** Meteorological conditions recorded during field trials.

**Table S4.** Comparison of different threshold methods for analysis of enzyme‐linked immunosorbent assay (ELISA) optical density results.

**Fig. S1.** The relative level of contamination of individuals within groups of naïve *Culicoides nubeculosus* exposed to different numbers of ovalbumin‐positive *C. nubeculosus*.

## References

[jpe12875-bib-0001] Akaike, H . (1973) Information theory as an extension of the Maximum likelihood principle 2nd International Symposium on Information Theory (eds Petrov B.N. & Csaksi F.), pp. 267–281. Akademiai Kiado, Budapest, Hungary.

[jpe12875-bib-0002] Alba, A. , Casal, J. & Domingo, M. (2004) Possible introduction of bluetongue into the Balearic Islands, Spain, in 2000, via air streams. Veterinary Record, 155, 460–461. 15518407 10.1136/vr.155.15.460

[jpe12875-bib-0003] Balenghien, T. , Pages, N. , Goffredo, M. *et al* (2014) The emergence of Schmallenberg virus across *Culicoides* communities and ecosystems in Europe. Preventive Veterinary Medicine, 116, 360–369. 24698329 10.1016/j.prevetmed.2014.03.007

[jpe12875-bib-0004] Biddinger, D.J. , Joshi, N.K. , Rajotte, E.G. , Halbrendt, N.O. , Pulig, C. , Naithani, K.J. & Vaughan, M. (2013) An immunomarking method to determine the foraging patterns of *Osmia cornifrons* and resulting fruit set in a cherry orchard. Apidologie, 44, 738–749.

[jpe12875-bib-0005] Bishop, A.L. , McKenzie, H.J. , Barchia, I.M. & Spohr, L.J. (2000) Moon phase and other factors affecting light‐trap catches of *Culicoides brevitarsis* Keiffer (Diptera: Ceratopogonidae). Australian Journal of Entomology, 39, 29–32.

[jpe12875-bib-0006] Brenner, R.J. , Wargo, M.J. , Stains, G.S. & Mulla, M.S. (1984) The dispersal of *Culicoides mohave* (Diptera: Ceratopogonidae) in the desert of Southern California. Mosquito News, 44, 343–350.

[jpe12875-bib-0007] Burgin, L. , Gloster, J. , Sanders, C. , Mellor, P. S. , Gubbins, S. & Carpenter, S . (2012) Investigating incursions of bluetongue virus using a model of long‐distance Culicoides biting midge dispersal. Transboundary and Emerging Diseases, 60, 263–272. 22672434 10.1111/j.1865-1682.2012.01345.x

[jpe12875-bib-0008] Campbell, M.M. & Kettle, D.S. (1976) Marking of adult *Culicoides brevitarsis* Kieffer (Diptera: Ceratopogonidae) *Australian* . Journal of Entomology, 14, 383–386.

[jpe12875-bib-0009] Campbell, J.A. & Pelham‐Clinton, E.C. (1960) Taxonomic review of the British species of *Culicoides* Latreille (Diptera, Ceratopogonidae). Proceedings of the Royal Entomological Society of London (B), 67, 181–302.

[jpe12875-bib-0010] Carpenter, S . (2001) Colonisation and dispersal studies of the Scottish biting midge, Culicoides impunctatus Goetghebuer. PhD thesis, University of Aberdeen.

[jpe12875-bib-0011] Carpenter, S. , Wilson, A. & Mellor, P.S. (2009) *Culicoides* and the emergence of bluetongue virus in northern Europe. Trends in Microbiology, 17, 172–178. 19299131 10.1016/j.tim.2009.01.001

[jpe12875-bib-0012] Carslaw, D.C. & Ropkins, K. (2012) openair – an R package for air quality data analysis. Environmental Modelling & Software, 27–28, 52–61.

[jpe12875-bib-0013] Dallas, J.F. , Cruickshank, R.H. , Linton, Y.M. *et al* (2003) Phylogenetic status and matrilineal structure of the biting midge, *Culicoides imicola*, in Portugal, Rhodes and Israel. Medical and Veterinary Entomology, 17, 379–387. 14651651 10.1111/j.1365-2915.2003.00454.x

[jpe12875-bib-0014] Davies, J.B. (1965) Three techniques for labelling *Culicoides* (Diptera: Heleidae) with radioactive tracers both in the laboratory and in the field. Mosquito News, 25, 419–422.

[jpe12875-bib-0015] Defra (2014) GB bluetongue virus disease control strategy, Department for Environment and Rural Affairs. Available at: https://www.gov.uk/government/uploads/system/uploads/attachment_data/file/343402/bluetongue-control-strategy-140727.pdf (accessed 24 October 2016).

[jpe12875-bib-0016] Dorie, V . (2014) blme: Bayesian Linear Mixed‐Effect Models, version 1.0‐2. Available at: http://cran.r-project.org/web/packages/blme/index.html (accessed 5 September 2016).

[jpe12875-bib-0017] Ducheyne, E. , de Deken, R. , Becu, S. , Codina, B. , Nomikou, K. , Mangana‐Vougiaki, O. , Georgiev, G. , Purse, B.V. & Hendrickx, G. (2007) Quantifying the wind dispersal of *Culicoides* species in Greece and Bulgaria. Geospatial Health, 1, 177–189. 18686243 10.4081/gh.2007.266

[jpe12875-bib-0018] Elbers, A.R.W. , Loeffen, W.L.A. , Quak, S. *et al* (2012) Seroprevalence of Schmallenberg virus antibodies among dairy cattle, the Netherlands, winter 2011‐2012. Emerging Infectious Diseases, 18, 1065–1071. 22709656 10.3201/eid1807.120323 PMC3376820

[jpe12875-bib-0019] Elbers, A.R.W. , Meiswinkel, R. , van Weezep, E. , van Oldruitenborgh‐Oosterbaan, M.M.S. & Kooi, E.A. (2013) Schmallenberg virus in *Culicoides* spp. biting midges, the Netherlands, 2011. Emerging Infectious Diseases, 19, 106–109. 23260040 10.3201/eid1901.121054 PMC3558002

[jpe12875-bib-0020] Graesboll, K. , Sumner, T. , Enoe, C. , Christiansen, L. E. & Gubbins, S . (2016) A comparison of dynamics in two models for the spread of a vector‐borne disease. Transboundary and Emerging Disease, 63, 215–223. 10.1111/tbed.12249 25056842

[jpe12875-bib-0021] Hagler, J.R. (2011) An immunological approach to quantify consumption of protein‐tagged *Lygus hesperus* by the entire cotton predator assemblage. Biological Control, 58, 337–345.

[jpe12875-bib-0022] Hagler, J.R. & Jackson, C.G. (1998) An immunomarking technique for labeling minute parasitoids. Environmental Entomology, 27, 1010–1016.

[jpe12875-bib-0023] Hagler, J.R. & Jackson, C.G. (2001) Methods for marking insects: current techniques and future prospects. Annual Review of Entomology, 46, 511–543. 10.1146/annurev.ento.46.1.511 11112178

[jpe12875-bib-0024] Hagler, J.R. & Jones, V.P. (2010) A protein‐based approach to mark arthropods for mark‐capture type research. Entomologia Experimentalis Et Applicata, 135, 177–192.

[jpe12875-bib-0025] Hagler, J.R. , Machtley, S.A. & Blackmer, F. (2015) A potential contamination error associated with insect protein mark‐capture data. Entomologia Experimentalis Et Applicata, 154, 28–34.

[jpe12875-bib-0026] Hagler, J.R. , Cohen, A.C. , Bradleydunlop, D. & Enriquez, F.J. (1992) New approach to mark insects for feeding and dispersal studies. Environmental Entomology, 21, 20–25.

[jpe12875-bib-0027] Hanski, I. , Alho, J. & Moilanen, A. (2000) Estimating the parameters of survival and migration of individuals in metapopulations. Ecology, 81, 239–251.

[jpe12875-bib-0028] Harrup, L.E. , Purse, B.V. , Golding, N. , Mellor, P.S. & Carpenter, S. (2013) Larval development and emergence sites of farm‐associated *Culicoides* in the United Kingdom. Medical and Veterinary Entomology, 27, 441–449. 23458570 10.1111/mve.12006

[jpe12875-bib-0029] Hendrickx, G. , Gilbert, M. , Staubach, C. , Elbers, A. , Mintiens, K. , Gerbier, G. & Ducheyne, E. (2008) A wind density model to quantify the airborne spread of *Culicoides* species during North‐Western Europe bluetongue epidemic, 2006. Preventive Veterinary Medicine, 87, 162–181. 18639355 10.1016/j.prevetmed.2008.06.009

[jpe12875-bib-0030] Hoffmann, B. , Bauer, B. , Bauer, C. *et al* (2009) Monitoring of putative vectors of bluetongue virus serotype 8, Germany. Emerging Infectious Diseases, 15, 1481–1484. 19788820 10.3201/eid1509.090562 PMC2819873

[jpe12875-bib-0031] Holbrook, F.R. , Belden, R.P. & Bobian, R.J. (1991) Rubidium for marking adults of *Culicoides variipennis* (Diptera: Ceratopogonidae). Journal of Medical Entomology, 28, 246–249. 2056505 10.1093/jmedent/28.2.246

[jpe12875-bib-0032] Hooper, K.R. & Woolson, E.A. (1991) Labeling a parasitic wasp, *Microplitis croceipes* (Hymenoptera: Braconidae), with trace elements for mark‐recapture studies. Annals of the Entomological Society of America, 84, 255–262.

[jpe12875-bib-0033] Jacquet, S. , Huber, K. , Pages, N. *et al* (2016) Range expansion of the bluetongue vector, *Culicoides imicola*, in continental France likely due to rare wind‐transport events. Scientific Reports, 6, 27247. 27263862 10.1038/srep27247 PMC4893744

[jpe12875-bib-0034] Jones, V.P. , Hagler, J.R. , Brunner, J.F. , Baker, C.C. & Wilburn, T.D. (2006) An inexpensive immunomarking technique for studying movement patterns of naturally occurring insect populations. Environmental Entomology, 35, 827–836.

[jpe12875-bib-0035] Kettle, D.S. (1960) The flight of *Culicoides impunctatus* Goetghebuer (Diptera: Ceratopogonidae) over moorland and its bearing on midge control. Bulletin of Entomological Research, 51, 461–490.

[jpe12875-bib-0036] Kirkeby, C. , Bodker, R. , Stockmarr, A. , Lind, P. & Heegaard, P.M. (2013) Quantifying dispersal of European *Culicoides* (Diptera: Ceratopogonidae) vectors between farms using a novel mark‐release‐recapture technique. PLoS ONE, 8, e61269. 23630582 10.1371/journal.pone.0061269 PMC3632603

[jpe12875-bib-0037] Kluiters, G. , Swales, H. & Baylis, M. (2015) Local dispersal of palaearctic *Culicoides* biting midges estimated by mark‐release‐recapture. Parasites & Vectors, 8, 86. 25886488 10.1186/s13071-015-0658-z PMC4327803

[jpe12875-bib-0038] Lillie, T.H. , Jones, R.H. & Marquardt, W.C. (1981) Microionized fluorescent dusts for marking *Culicoides varripennis* adults. Mosquito News, 41, 356–358.

[jpe12875-bib-0039] Lillie, T.H. , Kline, D.L. & Hall, D.W. (1985) The dispersal of *Culicoides mississipiensis* (Diptera: Ceratopogonidae) in a salt‐marsh near Yankeetown, Florida. Journal of the American Mosquito Control Association, 1, 463–467. 3880262

[jpe12875-bib-0040] Linhares, A.X. & Anderson, J.R. (1989) Dispersal of natural populations of *Culicoides variipennis* (Coquillett) (Diptera: Ceratopogonidae) in northern California. Bulletin of the Society for Vector Ecology, 14, 336–346.

[jpe12875-bib-0041] Linhares, A.X. & Anderson, J.R. (1990) The influence of temperature and moonlight on flight activity of *Culicoides variipennis* (Coquillett) (Diptera: Ceratopogonidae) in northern California. Pan‐Pacific Entomologist, 66, 199–207.

[jpe12875-bib-0042] Lysyk, T.J. & Axtell, R.C. (1986) Estimating numbers and survival of houseflies (Diptera:Muscidae) with mark‐recapture methods. Journal of Economic Entomology, 79, 1016–1022. 3745629 10.1093/jee/79.4.1016

[jpe12875-bib-0043] Manel, S. & Holderegger, R. (2013) Ten years of landscape genetics. Trends in Ecology & Evolution, 28, 614–621. 23769416 10.1016/j.tree.2013.05.012

[jpe12875-bib-0044] Nolan, D.V. , Carpenter, S. , Barber, J. , Mellor, P.S. , Dallas, J.F. , Mordue, A.J. & Piertney, S.B. (2007) Rapid diagnostic PCR assays for members of the *Culicoides obsoletus* and *Culicoides pulicaris* species complexes, implicated vectors of bluetongue virus in Europe. Veterinary Microbiology, 124, 82–94. 17478060 10.1016/j.vetmic.2007.03.019

[jpe12875-bib-0045] Purse, B.V. , Carpenter, S. , Venter, G.J. , Bellis, G. & Mullens, B.A. (2015) Bionomics of temperate and tropical *Culicoides* midges: knowledge gaps and consequences for transmission of *Culicoides*‐borne viruses. Annual Review of Entomology, 60, 373–392. 10.1146/annurev-ento-010814-020614 25386725

[jpe12875-bib-0046] R Development Core Team (2015) R: A Language and Environment for Statistical Computing. R Foundation for Statistical Computing, Vienna, Austria Available at: http://www.R-project.org (accessed 5th September 2016).

[jpe12875-bib-0047] RStudio Team (2015) RStudio: Integrated Development for R. RStudio, Inc., Boston, MA, USA Available at: http://www.rstudio.com/ (accessed 5 September 2016).

[jpe12875-bib-0048] Sanders, C.J. & Carpenter, S. (2014) Assessment of an immunomarking technique for the study of dispersal of *Culicoides* biting midges. Infection Genetics and Evolution, 28, 583–587. 10.1016/j.meegid.2014.01.020 24480050

[jpe12875-bib-0049] Sanders, C.J. , Harrup, L.E. , Tugwell, L.A. , Brugman, V.A. , England, M. & Carpenter, S. (2017) Data from: quantification of within‐ and between‐farm dispersal of Culicoides biting midges (Diptera: Ceratopogonidae) using an immunomarking technique. Dryad Digital Repository, 10.5061/dryad.s403s. PMC5655569 29104309

[jpe12875-bib-0050] Searle, K.R. , Blackwell, A. , Falconer, D. , Sullivan, M. , Butler, A. & Purse, B.V. (2013) Identifying environmental drivers of insect phenology across space and time: Culicoides in Scotland as a case study. Bulletin of Entomological Research, 103, 155–170. 22846228 10.1017/S0007485312000466

[jpe12875-bib-0051] Sedda, L. & Rogers, D. J . (2013) The influence of the wind in the Schmallenberg virus outbreak in Europe. Scientific Reports, 3, Article number 3361. 10.1038/srep03361 PMC6506448 24285292

[jpe12875-bib-0052] Sedda, L. , Brown, H.E. , Purse, B.V. , Burgin, L. , Gloster, J. & Rogers, D.J. (2012) A new algorithm quantifies the roles of wind and midge flight activity in the bluetongue epizootic in northwest Europe. Proceedings of the Royal Society B‐Biological Sciences, 279, 2354–2362. 10.1098/rspb.2011.2555 PMC3350672 22319128

[jpe12875-bib-0053] Sellers, R.F. (1980) Weather, host and vector – their interplay in the spread of insect borne animal virus diseases. Journal of Hygiene, 85, 65–102. 6131919 10.1017/s0022172400027108 PMC2134001

[jpe12875-bib-0054] Sellers, R.F. , Pedgley, D.E. & Tucker, M.R. (1978) Possible windborne spread of bluetongue to Portugal, June‐July 1956. Journal of Hygiene, 81, 189–196. 212475 10.1017/s0022172400025018 PMC2129786

[jpe12875-bib-0055] Sivakoff, F.S. , Rosenheim, J.A. & Hagler, J.R. (2011) Threshold choice and the analysis of protein marking data in long‐distance dispersal studies. Methods in Ecology and Evolution, 2, 77–85.

[jpe12875-bib-0056] Swezey, S.L. , Nieto, D.J. , Hagler, J.R. , Pickett, C.H. , Bryer, J.A. & Machtley, S.A. (2013) Dispersion, distribution, and movement of *Lygus* spp. (Hemiptera: Miridae) in trap‐cropped organic strawberries. Environmental Entomology, 42, 770–778. 23905741 10.1603/EN12353

[jpe12875-bib-0057] The Pirbright Institute (2007) Pictorial Guide to the Wings of British Culicodies (Diptera: Ceratopogonidae). Available at: www.Culicoides.net (accessed 5 February 2012).

[jpe12875-bib-0058] Venter, G.J. , Paweska, J.T. , Lunt, H. , Mellor, P.S. & Carpenter, S. (2005) An alternative method of blood‐feeding *Culicoides imicola* and other haematophagous *Culicoides* species for vector competence studies. Veterinary Parasitology, 131, 331–335. 15964690 10.1016/j.vetpar.2005.05.002

[jpe12875-bib-0059] Whiteman, C.D. & Doran, J.C. (1993) The relationship between overlying synoptic‐scale flows and winds within a valley. Journal of Applied Meteorology, 32, 1669–1682.

[jpe12875-bib-0060] Williams, R.W. (1962) Observations on the bionomics of *Culicoides furens* (Poey) on St John, U.S. Virgin Islands (Diptera: Ceratopogonidae). Mosquito News, 22, 155–157.

[jpe12875-bib-0061] Zeileis, A. , Kleiber, C. & Jackman, S. (2008) Regression models for count data in R. Journal of Statistical Software, 27, 1–25.

[jpe12875-bib-0062] Zimmerman, R.H. & Turner, E.C.J . (1984) Dispersal and gonotrophic age of *Culicoides variipennis* (Diptera: Ceratopogonidae). Journal of Medical Entomology, 21, 527–535. 6502612 10.1093/jmedent/21.5.527

