# Supplementary material for: Quantification of within‐ and between‐farm dispersal of Culicoides biting midges using an immunomarking technique
Source: J Appl Ecol. 2017 Feb 28;54(5):1429–39. doi: 10.1111/1365-2664.12875 (PMC5655569; doi:10.1111/1365-2664.12875)
Supplement: Supplementary file 1 — Table S1. Distance (m) of trap locations relative to the egg solution‐marked area and number of Culicoides collected. [file JPE-54-1429-s001.docx]

**Table S1. Distance (m) of ultraviolet (UV) miniature Centre for Disease Control (CDC) trap (model: 912, John W Hock, FL, USA) locations relative to the egg solution marked area and number of *Culicoides* collected split by *Culicoides* species/species group (number of females and males shown in parenthesis). Trap locations 15 to 24 only used in replicates three, four and six.**

| Trap Location | Distance from Egg Marked Area (m) | Bearing from Egg Marked Area (°) | Total *Culicoides* | Subgenus *Avaritia* *Culicoides* species† | *C. achrayi* Kettle and Lawson, 1955 | *C. albicans* (Winnertz), 1852 | *C. circumscriptus* Kieffer, 1918 | *C. chiopterus* (Meigen), 1830* | *C. clastrieri* Callot, Kremer and Deduit, 1962 | *C. dewulfi* Goetghebuer, 1936* | *C. festivipennis* Kieffer, 1914 | *C. nubeculosus* (Meigen), 1830 | *C. obsoletus* (Meigen), 1918* | *C. pictipennis* (Staeger), 1839 | *C. pulicaris* (L.), 1758 | *C. punctatus* (Meigen), 1804 | *C. scoticus* Downes and Kettle, 1952* | *C. riethi* Kieffer, 1914 |
| --- | --- | --- | --- | --- | --- | --- | --- | --- | --- | --- | --- | --- | --- | --- | --- | --- | --- | --- |
| 1  (*n* = 22) | 261.3 | 137.4 | 50  (48;2) | 17  (17;0) | 16  (16;0) | - | - | - | - | - | 6  (5;1) | 1  (1;0) | 2 (0;2) | 1  (1;0) | 4  (3;1) | 5  (5;0) | 2 (0;2) | - |
| 2  (*n* = 22) | 406.0 | 140.4 | 95  (94;1) | 10  (10;0) | 16  (16;0) | - | 1  (1;0) | - | - | - | 9  (8;1) | 1  (1;0) | 1 (0;1) | - | 52  (52;0) | 6  (6;0) | - | - |
| 3  (*n* = 22) | 697.7 | 149.8 | 582  (582;0) | 560  (560;0) | 1  (1;0) | - | 3  (3;0) | - | - | - | - | 2  (2;0) | - | - | 2  (2;0) | 14  (14;0) | - | - |
| 4  (*n* = 22) | 279.2 | 97.8 | 723  (720;3) | 464  (464;0) | 63  (63;0) | - | 1  (1;0) | - | - | 1 (0;1) | 30  (30;0) | 2  (2;0) | 6 (0;6) | 3  (3;0) | 46  (44;2) | 114  (113;1) | 4 (0;4) | - |
| 5  (*n* = 22) | 269.7 | 44.5 | 43  (40; 3) | 19  (17;2) | 15  (15;0) | - | - | - | - | - | 1  (0;1) | - | - | 1  (1;0) | 2  (2;0) | 5  (5;0) | 1 (0;1) | - |
| 6  (*n* = 22) | 307.0 | 3.6 | 299  (280;19) | 195  (178;17) | 24  (24;0) | - | 2  (2;0) | - | - | - | 17  (17;0) | - | - | 2  (1;1) | 16  (16;0) | 43  (42;1) | - | - |
| 7  (*n* = 22) | 247.3 | -12.4 | 856 (822;34) | 361  (336;25) | 111  (111;0) | - | - | 1  (0;1) | 1  (1;0) | - | 15  (15;0) | - | 2 (0;2) | 3  (3;0) | 166  (163;3) | 199  (193;6) | 2 (0;2) | - |
| 8  (*n* = 22) | 246.6 | -48.1 | 513  (507;6) | 340  (338;2) | 27  (27;0) | - | 1  (1;0) | - | - | 1 (0;1) | 64  (62;2) | - | 1 (0;1) | - | 42  (41;1) | 39  (38;1) | - | - |
| 9  (*n* = 22) | 6.3 | -43.0 | 3220  (3216;4) | 3084  (3081;3) | 3  (3;0) | - | 7  (7;0) | 2  (0;2) | - | 6 (0;6) | - | 1  (0;1) | 32 (0;32) | - | 43  (43;0) | 82  (82;0) | 15 (0;15) | - |
| 10  (*n* = 22) | 8.7 | 125.1 | 298  (296;2) | 290  (288;2) | 1  (1;0) | - | - | - | - | 1 (0;1) | 2  (2;0) | - | 26 (1;25) | - | 2  (2;0) | 3  (3;0) | 16 (0;16) | - |
| 11  (*n* = 22) | 203.7 | -132.1 | 486  (474;12) | 160  (157;3) | 75  (75;0) | - | 1  (1;0) | - | - | - | 25  (25;0) | - | 4 (0;4) | 1  (1;0) | 85  (77;8) | 139  (138;1) | 1 (0;1) | - |
| 12  (*n* = 22) | 248.0 | -159.8 | 99 (99;0) | 27  (27;0) | 27  (27;0) | - | 1  (1;0) | - | 1  (1;0) | 2 (0;2) | 3  (3;0) | - | 2 (0;2) | 1  (1;0) | 8  (8;0) | 32  (32;0) | 3 (0;3) | - |
| 13  (*n* = 22) | 314.3 | -177.4 | 374  (372;2) | 62  (62;0) | 196  (196;0) | - | 1  (1;0) | 2  (0;2) | - | 6 (0;6) | 30  (29;1) | - | 17 (0;17) | 5  (5;0) | 27  (27;0) | 52  (51;1) | 14 (0;14) | - |
| 14  (*n* = 22) | 304.3 | 165.2 | 139  (133; 6) | 37  (35;2) | 5  (5;0) | - | 1  (1;0) | - | - | - | 51  (50;1) | - | 1 (0;1) | 1  (1;0) | 13  (11;2) | 31  (30;1) | 3 (0;3) | - |
| 15  (*n* = 12) | 650.6 | 77.9 | 1  (1;0) | 1  (1;0) | - | - | - | - | - | - | - | - | 1 (0;1) | - | - | - | - | - |
| 16  (*n* = 12) | 1543.9 | 68.8 | 3  (3;0) | 2  (2;0) | - | - | - | - | - | - | 1  (1;0) | - | 1 (0;1) | - | - | - | 1 (0;1) | - |
| 17  (*n* = 12) | 2541.9 | 66.2 | 56  (56;0) | 42  (42;0) | 1  (0;0) | 1  (1;0) | - | 2  (0;2) | - | 3 (0;3) | - | 5  (5;0) | 26 (0;26) | - | 5  (5;0) | 1  (1;0) | 11 (0;11) | 1  (0;1) |
| 18  (*n* = 12) | 3875.6 | 71.6 | 6  (6;0) | 5  (5;0) | 1  (1;0) | - | - | - | - | - | - | - | 2 (0;2) | - | - | - | 3 (0;3) | - |
| 19  (*n* = 12) | 3125.0 | 88.9 | 317  (317;0) | 267  (267;0) | 15  (15;0) | - | - | 7  (0;7) | - | 26 (0;26) | 3  (3;0) | - | 134 (0;134) | - | 29  (29;0) | 3  (3;0) | 100 (0;100) | - |
| 20  (*n* = 12) | 1926.4 | 85.6 | 18  (18;0) | 15  (15;0) | 2  (2;0) | - | - | - | - | 1 (0;1) | 1  (1;0) | - | 10 (0;10) | - | - | - | 4 (0;4) | - |
| 21  (*n* = 12) | 1384.8 | 94.6 | 423  (423;0) | 340  (340;0) | 7  (7;0) | - | - | 6  (0;6) | - | 36 (0;36) | - | - | 175 (0;175) | - | 76  (76;0) | - | 123 (0;123) | - |
| 22  (*n* = 12) | 710.1 | -144.0 | 402  (402;0) | 357  (357;0) | 19  (19;0) | - | - | 13 (0;13) | - | 27 (0;27) | 1  (1;0) | - | 196 (0;196) | - | 13  (13;0) | 12  (12;0) | 121 (0;121) | - |
| 23  (*n* = 12) | 1377.1 | -136.9 | 43  (43;0) | 33  (33;0) | - | - | - | - | - | 6  (0;6) | - | - | 11 (0;11) | - | 10  (10;0) | - | 16 (0;16) | - |
| 24  (*n* = 12) | 2132.4 | -127.8 | 12  (12;0) | 12  (12;0) | - | - | - | - | - | 1  (0;1) | - | - | 7  (0;7) | - | - | - | 4  (0;4) | - |
| † Includes number of *Culicoides* displayed in *C. chiopterus*, *C. dewulfi, C. obsoletus* and *C. scoticus* columns, number collected in replicates 1-5  * Species identifications based on multiplex PCR assay, number collected in replicates 3, 4 and 5 only | | | | | | | | | | | | | | | | | | |
