# Supplementary material for: Quantification of within‐ and between‐farm dispersal of Culicoides biting midges using an immunomarking technique
Source: J Appl Ecol. 2017 Feb 28;54(5):1429–39. doi: 10.1111/1365-2664.12875 (PMC5655569; doi:10.1111/1365-2664.12875)
Supplement: Supplementary file 2 — Table S2. Number of Culicoides collected positive for ovalbumin during immune‐marking studies. [file JPE-54-1429-s002.docx]

**Table S2. Number of *Culicoides* collected positive for ovalbumin during immune-marking studies (number of females and males respectively shown in parenthesis) split trap location and *Culicoides* species/species group.**

| Trap Location | | Distance from Egg Marked Area (m) | Bearing from Egg Marked Area (°) | Total *Culicoides* | Subgenus *Avaritia* *Culicoides* species† | *C. achrayi* Kettle and Lawson, 1955 | *C. albicans* (Winnertz), 1852 | *C. chiopterus* (Meigen), 1830* | *C. circumscriptus* Kieffer, 1918 | *C. clastrieri* Callot, Kremer and Deduit, 1962 | *C. dewulfi* Goetghebuer, 1936* | *C. festivipennis* Kieffer, 1914 | *C. nubeculosus* (Meigen), 1830 | *C. obsoletus* (Meigen), 1918* | *C. pictipennis* (Staeger), 1839 | *C. pulicaris* (L.), 1758 | *C. punctatus* (Meigen), 1804 | *C. scoticus* Downes and Kettle, 1952* | *C. riethi* Kieffer, 1914 |
| --- | --- | --- | --- | --- | --- | --- | --- | --- | --- | --- | --- | --- | --- | --- | --- | --- | --- | --- | --- |
| 1  (*n* = 22) | | 261.3 | 137.4 | 11  (1;10) | 1  (0;1) | 4  (0;4) | - | - | - | - | - | 3  (1;2) | 1  (0;1) | 1  (0;1) | - | - | 2  (0;2) | - | - |
| 2  (*n* = 22) | | 406.0 | 140.4 | 21  (1;20) | - | - | - | - | 1  (0;1) | - | - | 2  (1;1) | - | - | - | 15  (0;15) | 3  (0;3) | - | - |
| 3  (*n* = 22) | | 697.7 | 149.8 | 4  (0;4) | 3  (0;3) | - | - | - | - | - | - | - | 1  (0;1) | - | - | - | - | - | - |
| 4  (*n* = 22) | | 279.2 | 97.8 | 28  (0;28) | 9  (0;9) | - | - | - | 1  (1;0) | - | - | 5  (0;5) | - | 5  (0;5) | - | 2  (0;2) | 11  (0;11) | - | - |
| 5  (*n* = 22) | | 269.7 | 44.5 | 4  (0;4) | - | 1  (0;1) | - | - | - | - | - | - | - | - | - | 1  (0;1) | 2  (0;2) | - | - |
| 6  (*n* = 22) | | 307.0 | 3.6 | 27  (2;25) | 10  (2;8) | - | - | - | - | - | - | 7  (0;7) | - | - | - | 4  (0;4) | 6  (0;6) | - | - |
| 7  (*n* = 22) | | 247.3 | -12.4 | 116  (4;112) | 7  (2;5) | 16  (0;16) | - | - | - | - | - | 4  (0;4) | - | - | - | 40  (1;39) | 49  (0;49) | - | - |
| 8  (*n* = 22) | | 246.6 | -48.1 | 36  (0;36) | 3  (0;3) | - | - | - | - | - | 1  (0;1) | 21  (0;21) | - | - | - | 8  (0;8) | 4  (0;4) | - | - |
| 9  (*n* = 22) | | 6.3 | -43.0 | 142  (1;141) | 92  (0;92) | - | - | - | 4  (0;4) | - | 1  (0;1) | - | 1  (1;0) | - | - | 19  (0;19) | 26  (0;26) | 1  (0;1) | - |
| 10  (*n* = 22) | | 8.7 | 125.1 | 5  (0;5) | 3  (0;3) | 1  (0;1) | - | - | - | - | - | - | - | - | - | 1  (0;1) | - | - | - |
| 11  (*n* = 22) | | 203.7 | -132.1 | 74  (3;71) | 6  (0;6) | 13  (0;13) | - | - | - | - | - | 10  (0;10) | - | - | - | 19  (3;16) | 26  (0;26) | - | - |
| 12  (*n* = 22) | | 248.0 | -159.8 | 15  (0;15) | 1  (0;1) | 2  (0;2) | - | - | - | - | - | 1  (0;1) | - | - | 1  (0;1) | 2  (0;2) | 8  (0;8) | 1  (0;1) | - |
| 13  (*n* = 22) | | 314.3 | -177.4 | 52  (1; 51) | 1  (0;1) | 25  (0;25) | - | - | - | - | - | 9  (1;8) | - | 1  (0;1) | 4  (0;4) | 2  (0;2) | 11  (0;11) | - | - |
| 14  (*n* = 22) | | 304.3 | 165.2 | 26  (1;25) | 2  (0;2) | 1  (0;1) | - | - | - | - | - | 14  (0;14) | - | - | - | 4  (0;4) | 5  (1;4) | - | - |
| 15  (*n* = 12) | | 650.6 | 77.9 | - | - | - | - | - | - | - | - | - | - | - | - | - | - | - | - |
| 16  (*n* = 12) | | 1543.9 | 68.8 | - | - | - | - | - | - | - | - | - | - | - | - | - | - | - | - |
| 17  (*n* = 12) | | 2541.9 | 66.2 | 4  (0;4) | 1  (0;1) | - | - | - | - | - | - | - | 2  (0;2) | - | - | 1  (0;1) | - | 1  (0;1) | - |
| 18  (*n* = 12) | | 3875.6 | 71.6 | - | - | - | - | - | - | - | - | - | - | - | - | - | - | - | - |
| 19  (*n* = 12) | | 3125.0 | 88.9 | 17  (0;17) | 14  (0;14) | - | - | 1  (0;1) | - | - | 3  (0;3) | - | - | 6  (0;6) | - | 3  (0;3) | - | 4  (0;4) | - |
| 20  (*n* = 12) | | 1926.4 | 85.6 | 1  (0;1) | 1  (0;1) | - | - | - | - | - | - | - | - | 1  (0;1) | - | - | - | - | - |
| 21  (*n* = 12) | | 1384.8 | 94.6 | 8  (0;8) | 5  (0;5) | - | - | - | - | - | - | - | - | 2  (0;2) | - | 3  (0;3) | - | 3  (0;3) | - |
| 22  (*n* = 12) | | 710.1 | -144.0 | 7  (0;7) | 6  (0;6) | - | - | - | - | - | - | - | - | 4  (0;4) | - | - | 1  (0;1) | 2  (0;2) | - |
| 23  (*n* = 12) | | 1377.1 | -136.9 | 2  (0;2) | 2  (0;2) | - | - | - | - | - | 1  (0;1) | - | - | - | - | - | - | 1  (0;1) | - |
| 24  (*n* = 12) | | 2132.4 | -127.8 | - | - | - | - | - | - | - | - | - | - | - | - | - | - | - | - |
| † Includes number of *Culicoides* displayed in *C. chiopterus*, *C. dewulfi, C. obsoletus* and *C. scoticus* columns, number collected in replicates 1-5  * Species identifications based on multiplex PCR assay, number collected in replicates 3, 4 and 5 only | | | | | | | | | | | | | | | | | | | |
