# Supplementary material for: Quantification of within‐ and between‐farm dispersal of Culicoides biting midges using an immunomarking technique
Source: J Appl Ecol. 2017 Feb 28;54(5):1429–39. doi: 10.1111/1365-2664.12875 (PMC5655569; doi:10.1111/1365-2664.12875)
Supplement: Supplementary file 3 — Table S3. Meteorological conditions recorded during field trials. [file JPE-54-1429-s003.docx]

# Table S3. Meteorological conditions recorded during field trials split by trapping session (- indicates data not available).

| Replicate | Day | Trapping Session | Precipitation  (mm) | | Relative Humidity  (%) | | Air Temperature  (°C) | | Solar Radiation  (W/m^2^) | | Windspeed  (m/s) | | Maximum Gust Speed  (m/s) | | Wind Direction  (°) | | Lunar Illumination  (%) |
| --- | --- | --- | --- | --- | --- | --- | --- | --- | --- | --- | --- | --- | --- | --- | --- | --- | --- |
|  |  |  | Mean  (Min;Max) | SD | Mean  (Min;Max) | SD | Mean  (Min;Max) | SD | Mean  (Min;Max) | SD | Mean  (Min;Max) | SD | Mean  (Min;Max) | SD | Mean  (Min;Max) | SD |  |
| 1 | 1 | 1 | 0  (0;0) | 0 | 49.7  (34.4;64.3) | 10.4 | 13.4  (7.9;21.3) | 4.1 | 86.9  (0;436.1) | 131 | 1  (0;2.8) | 0.8 | - | - | 270.2  (0;320.6) | 67 | 23 |
|  | 2 | 2 | 0  (0;0) | 0 | 42.5  (32.4;64.6) | 11.3 | 14.5  (11;20.7) | 2.7 | 58.7  (0;430.6) | 100.7 | 2.3  (0.8;3.9) | 0.8 | - | - | 289.7  (277.7;322.2) | 12.1 | 14 |
|  | 3 | 3 | 0  (0;0) | 0 | 42.3  (33.3;64.4) | 12 | 13.7  (10.7;20.5) | 3.3 | 54.2  (0;423.7) | 100.1 | 1.7  (0.8;2.5) | 0.5 | - | - | 285.8  (275.6;307.2) | 9.4 | 9 |
| 2 | 1 | 4 | 0  (0;0) | 0 | 44.1  (30.8;69.2) | 16.1 | 17.8  (15.2;24.1) | 2.9 | 55.7  (0;411.2) | 101.9 | 3.3  (0.2;6.4) | 1 | 5.1  (0.6;10) | 1.5 | 59.7  (0.2;121.3) | 14.9 | 98 |
|  | 2 | 5 | 0  (0;1.6) | 0.2 | 36.7  (29;67.3) | 10.6 | 19.7  (15.9;28.7) | 3.3 | 33.7  (0;270.2) | 67.2 | 1.4  (0;5.8) | 1.1 | 2.1  (0;9) | 1.7 | 161.6  (0.3;359.2) | 75.2 | 100 |
|  | 3 | 6 | 0  (0;0) | 0 | 42.3  (30.6;65) | 13 | 16.7  (12.2;25.6) | 3.8 | 50.4  (0;398.7) | 86.4 | 1.3  (0;7.2) | 1.4 | 2  (0;11.1) | 2.2 | 202.5  (119.5;357.2) | 51 | 99 |
|  | 4 | 7 | 0.1  (0;1.2) | 0.3 | 44  (30;67) | 15.3 | 17.8  (15.8;24.1) | 2.3 | 37.6  (0;321.7) | 76.1 | 2.3  (0;7.7) | 1.4 | 3.5  (0;11.2) | 2.2 | 188.8  (7.3;356.2) | 49 | 96 |
| 3 | 1 | 8 | 0  (0;0) | 0 | 41.9  (34.8;66.6) | 9.4 | 12.8  (7.4;21.9) | 4.3 | 35.6  (0;348) | 71.8 | 0.9  (0;5.4) | 1.2 | 1.4  (0;8.7) | 1.7 | 139.9  (0.1;355.5) | 105.3 | 59 |
|  | 2 | 9 | 0  (0;0.2) | 0 | 41.5  (32.2;65.2) | 11.3 | 14.2  (9;23.3) | 3.9 | 54.7  (0;430.8) | 104 | 1  (0;6.5) | 1.3 | 1.5  (0;10.4) | 1.8 | 249.4  (62.8;353.7) | 73 | 49 |
|  | 3 | 10 | 0  (0;0) | 0 | 43.3  (30.2;65.7) | 12.9 | 16.5  (12.6;21.9) | 2.3 | 27  (0;369.5) | 62.2 | 1.8  (0;6.4) | 1.2 | 3.3  (0;10.7) | 2.1 | 246.2  (121.3;325.3) | 41 | 40 |
| 4 | 1 | 11 | 0  (0;0) | 0 | 0.7  (0.5;0.9) | 0.1 | 13.8  (11.1;20.7) | 3 | 25.4  (0;208.7) | 41.7 | 1  (0;2.6) | 0.5 | 1.4  (0.1;3.5) | 0.7 | 79.1  (0;358) | 95 | 0 |
|  | 2 | 12 | 0  (0;0) | 0 | 0.8  (0.6;0.9) | 0.1 | 17.3  (13.6;22.8) | 2.6 | 27.6  (0;377.2) | 49.3 | 1.1  (0;4.4) | 0.8 | 1.6  (0.1;6.4) | 1.1 | 179.3  (0;356) | 72 | 0 |
|  | 3 | 13 | 0  (0;0) | 0 | 0.8  (0.5;0.9) | 0.1 | 12.9  (7.2;20.2) | 3.5 | 59.7  (0;393.7)* | 102.1 | 0.7  (0;2.7) | 0.5 | 1.3  (0.1;5.6) | 0.9 | 233.6  (0;358) | 77.2 | 2 |
|  | 4 | 14 | 0  (0;0.2) | 0 | 0.8  (0.5;0.9) | 0.1 | 14.9  (12.2;20.7) | 2.4 | 38.5  (0;499.9) | 75.6 | 0.7  (0;3) | 0.4 | 1.1  (0.1;4.8) | 0.7 | 241.4  (0;358) | 47.9 | 6 |
| 5 | 1 | 15 | 0  (0;0) | 0 | 0.8  (0.6;1) | 0.1 | 12.3  (8.1;20.9) | 4 | 27.7  (0;203.2) | 45.5 | 0.6  (0;3) | 0.5 | 0.9  (0.1;5) | 0.7 | 213.2  (0;358) | 98.2 | 16 |
|  | 2 | 16 | 0  (0;0) | 0 | 0.9  (0.7;0.9) | 0.1 | 13.7  (10.9;18) | 2.2 | 29.4  (0;283.8) | 50 | 0.3  (0;2.2) | 0.3 | 0.5  (0;3) | 0.5 | 174.2  (0;358) | 134.4 | 9 |
|  | 3 | 17 | 0  (0;0.4) | 0 | 0.8  (0.6;0.9) | 0.1 | 16.4  (13.8;22.3) | 2.6 | 22.5  (0;327.8) | 48.3 | 1.5  (0.2;4) | 0.6 | 2.4  (0.3;6.5) | 1 | 137.6  (26;197) | 32.8 | 4 |
|  | 4 | 18 | 0  (0;1) | 0.1 | 0.9  (0.8;0.9) | 0 | 16.4  (15.1;19.1) | 1.3 | 12.9  (0;130) | 26.8 | 1  (0.1;2.9) | 0.4 | 1.4  (0.1;4.3) | 0.7 | 93.7  (22;187) | 30.6 | 1 |
| 6 | 1 | 19 | 0  (0;1.4) | 0.1 | 0.9  (0.6;1) | 0.1 | 9.6  (5.6;18.5) | 3.1 | 29.3  (0;509) | 64.7 | 0.3  (0;3.6) | 0.4 | 0.5  (0;6.1) | 0.6 | 187.8  (0;358) | 113.2 | 2 |
|  | 2 | 20 | 0  (0;0) | 0 | 0.8  (0.6;0.9) | 0.1 | 11.2  (7.4;17.7) | 2.6 | 28.7  (0;604.2) | 56.9 | 0.8  (0;3.7) | 0.8 | 1.3  (0.1;5.5) | 1 | 255.4  (0;358) | 114.3 | 3 |
|  | 3 | 21 | 0  (0;0) | 0 | 0.8  (0.6;0.8) | 0 | 11.5  (10.5;14) | 1 | 20.8  (0;349.7) | 47.5 | 0.9  (0;4) | 0.7 | 1.4  (0.1;5.5) | 0.9 | 263.1  (0;358) | 129.3 | 4 |
|  | 4 | 22 | 0  (0;0) | 0 | 0.8  (0.6;0.9) | 0.1 | 11.6  (7.8;16.2) | 2.1 | 27.2  (0;468.8) | 54.2 | 0.9  (0;4.6) | 0.9 | 1.3  (0.1;6.3) | 1.2 | 186.2  (0;358) | 133.9 | 5 |
