# Supplementary material for: Quantification of within‐ and between‐farm dispersal of Culicoides biting midges using an immunomarking technique
Source: J Appl Ecol. 2017 Feb 28;54(5):1429–39. doi: 10.1111/1365-2664.12875 (PMC5655569; doi:10.1111/1365-2664.12875)
Supplement: Supplementary file 4 — Table S4. Comparison of different threshold methods for analysis of enzyme‐linked immunosorbent assay (ELISA) optical density results. [file JPE-54-1429-s004.docx]

Table S4. **Comparison of different threshold methods for analysis of enzyme-linked immunosorbent assay (ELISA) optical density results (classical threshold as proposed by Stimmann (1974) with plate‑by‑plate variation accounted for as per Clark and Adams (1977); standard normal variate (SNV) transformation with maximum negative control threshold as proposed by Sivakoff, Rosenheim and Hagler (2011)).**

| Performance Measure | | Classical Threshold  $T=\mu+3s_{j}$ | Standard Normal Variate Transformation (SNV)  $z_{ij}=\frac{(X_{ij}- \hat{\mu}_{j})}{c\hat{\mu}_{j}^{k}}$ |
| --- | --- | --- | --- |
| True Positive rate (*TP*) | $=\frac{TP}{(TP+TN)}$ | 0.85 | 0.78 |
| True Negative Rate (*TN*) | $=\frac{TN}{(TN+FP)}$ | 1.00 | 1.00 |
| False Positive Rate (*FP*) | $=\frac{FP}{(TN+FP)}$ | 0.00 | 0.00 |
| False Negative Rate (*FN*) | $=\frac{FN}{(TP+FN)}$ | 0.15 | 0.22 |
| Maximum estimated dispersal distance | | 3, 125m | 3,125m |
| Number of trap locations (*n = 24)* at which ovalbumin-positive insects collected | | 22 | 20 |
| Proportion of total trap catch ovalbumin-positive | | 27.1% | 6.8% |
