# Supplementary material for: Quantification of within‐ and between‐farm dispersal of Culicoides biting midges using an immunomarking technique
Source: J Appl Ecol. 2017 Feb 28;54(5):1429–39. doi: 10.1111/1365-2664.12875 (PMC5655569; doi:10.1111/1365-2664.12875)
Supplement: Supplementary file 5 — Fig. S1. The relative level of contamination of individuals within groups of naïve Culicoides nubeculosus exposed to different numbers of ovalbumin‐positive C. nubeculosus. [file JPE-54-1429-s005.docx]

**Figure S1. The relative level of contamination of individuals within groups of 20 or 50 naïve *Culicoides nubeculosus* exposed to different numbers (1, 2, 5, 10 or 15) of ovalbumin-positive *C. nubeculosus* seeded into trap collection pots.** *C. nubeculosus* were marked by exposure in cardboard, mesh-covered pots previously sprayed with a fine mist of 20 % (*w/v*) egg white solution. After three hours the potentially marked *Culicoides* were removed and added to a clean trap collection pot with paper substrate as containing either a low population of 20 unmarked *C. nubeculosus* (left) or a higher population of 50 unmarked *C. nubeculosus* (right)*.* The marked and unmarked *Culicoides* were allowed to mix at room temperature within the new collection pot for nine hours to simulate the temporal and spatial conditions that insects would experience upon collection in the field study. All *Culicoides* were then killed by exposure to cold and stored at -20°C prior to assessment for the presence of ovalbumin marker on individual *Culicoides* using the ovalbumin-specific ELISA. Three replicates were performed at each density and number of marked individuals introduced.. The relative level of transfer from marked to unmarked *Culicoides* was calculated from comparison of the observed and expected number of ovalbumin-positive individuals recovered from each pot. In one replicate at the high density of insects the number of positive individuals exceeded the number introduced, indicating a probability of transfer of the marker between individuals at high densities of marked *Culicoides*. **
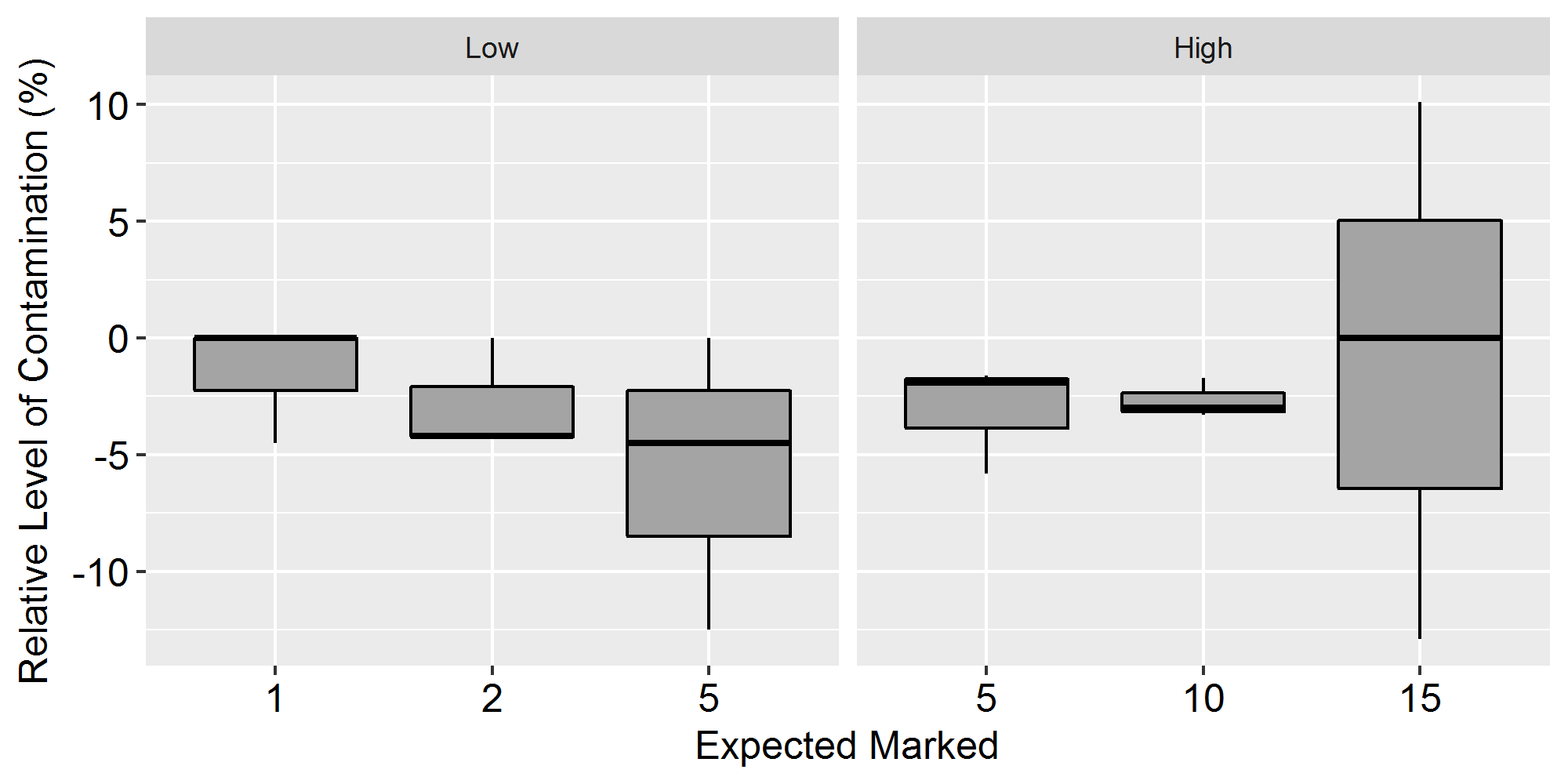
**
